# The role of microvesicles as biomarkers in the screening of colorectal neoplasm

**DOI:** 10.1002/cam4.4664

**Published:** 2022-03-27

**Authors:** Mohammad M. R. Eddama, Rijan Gurung, Konstantinos Fragkos, Paula Lorgelly, Richard Cohen, Marilena Loizidou, Lucie Clapp

**Affiliations:** ^1^ Research Department of Surgical Biotechnology, Division of Surgery and Interventional Science University College London London UK; ^2^ Department of Surgery University College London Hospital London UK; ^3^ Department of Gastroenterology University College London Hospital London UK; ^4^ Department of Applied Health Research Institute of Epidemiology and Health, University College London London UK; ^5^ Institute of Cardiovascular Sciences University College London London UK

**Keywords:** benign colorectal polyps, bowel screening, colorectal cancer, microvesicles

## Abstract

**Background:**

Colorectal cancer (CRC) is the second cause of cancer death worldwide. The role of circulating microvesicles as a screening tool is a novel, yet effective approach that warrants prioritised research.

**Methods:**

In a two‐gate diagnostic accuracy study, 35 patients with benign colorectal polyps (BCRP) (*n* = 16) and colorectal cancer (CRC) (*n* = 19) were compared to 17 age‐matched healthy controls. Total annexin‐V positive microvesicles and sub‐populations positive for selected biomarkers relevant to bowel neoplasm were evaluated in patients' plasma using flow cytometry. Statistical methods including factor analysis utilising two component factors were performed to obtain optimal diagnostic accuracy of microvesicles in identifying patients with colorectal neoplasms.

**Results:**

Total plasma microvesicles, and sub‐populations positive for CD31, CD42a, CD31+/CD42a‐, EPHB2, ICAM and LGR5 (component factor‐1) were able to identify patients with BCRP and CRC with a receiver operator curve (AUC) accuracy of a 100% (95% CI: 100%–100%) and 95% (95% CI: 88%–100%), respectively. To identify patients with BCRP, a cut‐off point value of component factor‐1761 microvesicles/μl demonstrated a 100% sensitivity, specificity and negative predictive value (NPV) and a 93% positive predictive value (PPV). To identify patients with CRC, a cut‐off value of component factor‐1 ^3^439 microvesicles/μl demonstrated a 100% sensitivity, specificity and NPV and a 65% PPV. CEA+ microvesicles sub‐population were significantly (*p* < 0.02) higher in CRC in comparison to BCRP.

**Conclusions:**

Microvesicles as biomarkers for the early and accurate detection of CRC is a simple and effective tool that yields a potential breakthrough in clinical management.

## Graphical abstract

Microvesicles plasma concentration is significantly high in patients suffering from benign and malignant colorectal neoplasm. The use of microvesicles as biomarkers could improve bowel cancer screening programs utility.

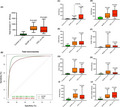

**Lay summary**

We found that plasma microvesicles level provides a high predictive value for benign and malignant colorectal neoplasm. The clinical implications of our findings are extensive in that microvesicles as a screening tool could improve the compliance and utility of the current bowel cancer screening faecal immunochemical test. A small volume of 100 μl of platelet poor plasma is sufficient to perform a microvesicle test, which can potentially be sampled from finger‐prick capillary blood. As a screening tool microvesicles could identify patients suffering from colorectal neoplasm and possibly other health conditions early before symptoms occur.

## 1 INTRODUCTION

According to the World Health Organization (WHO), cancer is the second leading cause of death globally. Colorectal cancer (CRC) is the second most common cause of cancer death, after lung cancer worldwide. The incidence of CRC has increased by 9.5% between 1990 and 2017, not only in high‐income countries, but also in most middle‐ and low‐income countries.^1^ What was known to be the disease of older generation is now affecting more younger people,^2^ possibly due to urbanisation, westernised lifestyle and risk factors, such as alcohol and unhealthy food consumption, obesity and smoking.^3^ Furthermore, the catastrophic impact of the COVID‐19 pandemic on healthcare resources is becoming increasingly evident. Indeed, due to the pandemic the ‘hidden backlog’ of people requiring cancer diagnosis and treatment may take years to rectify.^4^ We are in urgent need for a solution to mitigate these challenges. One of the strategic solutions to fight cancer in general and increase the chances of cure is by early detection. This strategy would not only reduce mortality, but also would significantly improve quality of life. James Lind Alliance Priority Setting Partnerships lists early detection of cancer as a top research priority. In the UK, the bowel cancer screening programme, using both stool‐based faecal sample and flexible sigmoidoscopy (Figure S1) for a specified population, has already reduced mortality by approximately 20%.^5^ However, there are two main limitations to the current stool‐sampling screening test. First, its predictive value is low, resulting in many patients undergoing unnecessary invasive investigations.^6^ Second, the compliance rate for the faecal immunochemical test (FIT) remains low (60%)^7^, with participants finding stool sampling challenging.^8^ To address this, the role of microvesicles (MVs) as biomarkers for bowel screening is a novel, yet promising approach that warrants prioritised research to ascertain its feasibility for clinical application.

MVs are small (~150–1000 nm) membranous sacs released from cells when they undergo proliferation, cell division or apoptosis.^9^ When initially discovered in 1976,^10^ MVs were believed to be cellular debris and pro‐coagulant dust that came from activated platelets. Here, we briefly describe MVs biogenesis and molecular cargo. MVs are one of the three types of extracellular vesicles (EVs) distinguished by their size and biogenesis. The other two main types of EVs are exosomes and apoptotic bodies. Exosomes are smaller (<150 nm) and derive from multivesicular bodies within the cell's endosomal system. Apoptotic bodies are larger (>1000 nm) and arise from dying cells.^11^ MVs' bioactive cargo resembles the molecular composition of the original cell, such as membrane receptors, cytoskeletal components, messenger RNA (mRNA), microRNAs (miRNA) and cytoplasmic DNA.^12^ MVs enter the circulation and can be isolated and employed as surrogate biomarkers.^13^ In all probability and in the context of neoplasia MVs are a molecular biopsy from tumorous cells. The idea of employing MVs as biomarkers is new, exciting and feasible.

Biomarkers with potential roles in tumorigenesis and progression, such as carcinoembryonic antigen (CEA),^14^ ephrin‐type‐B receptor 2 (EPHB2),^15^ intracellular adhesion molecule 1 (ICAM‐1 or CD54),^16^ leucine‐rich repeat containing G protein‐coupled receptor 5 (LGR5),^17^ glycoprotein A33 antigen (A33),^18^ platelet endothelial cell adhesion molecule (PECAM‐1), also known as CD31,^19^ and platelet membrane glycoprotein IX, also known as CD42a,^20^ may demonstrate a useful role in early CRC detection. For example, the serum level of CEA is currently used to monitor therapy and recurrence, however, due to poor sensitivity and specificity it is unreliable for early detection of patients with colorectal neoplasm.^21^ Yet, we do not know the expression of CEA and other biomarkers in MVs in patients suffering from colorectal neoplasm.

A major difficulty in conducting studies of biomarkers in relation to CRC is the high degree of correlation among various markers. We anticipated similar situation in our study. Isolating the effects of an individual biomarker becomes a serious methodologic problem. Moreover, the assumption that individual biomarkers have isolated effects may not be valid. In this context, factor analysis has been used as a variable reduction technique to deal with this information issue.^22^ Factor analysis is a variable consolidation technique designed to generate a small number of variables that will capture much of the information in a larger data set. In this way, factor analysis allows an investigator to reduce information on the various biomarkers into 2 or 3 variables that capture the primary sources of variation in the reported biomarker panel. In this study we plan utilise factor analysis so that the predictive value of these biomarkers for colorectal neoplasia is optimised. Our objective is to assess the feasibility and potential predictive value of total plasma MVs and their subpopulations for identifying patients with colorectal neoplasia, including adenomatous benign colorectal polyps (BCRP) and CRC.

## 2 MATERIALS AND METHODS

### 2.1 Patients and sample collection

In a two‐gate diagnostic accuracy study, we enrolled patients presenting to the 2‐week wait colorectal target clinic with symptoms including rectal bleeding, change in bowel habit, iron deficiency anaemia and weight loss. Additionally, patients with established diagnoses of bowel cancer were included as positive controls. All samples including routine blood tests and plasma for MVs isolation were collected from patients at the same time. Samples were collected from CRC patients before receiving cancer treatment. All patients included in this study attended University College London Hospital between October 2017 and October 2018 and had confirmed endoscopic and histologic diagnosis of colorectal neoplasm. A total of 35 patients with BCRP (*n* = 16) and CRC (*n* = 19), were included alongside age‐matched healthy control participants (*n* = 17) recruited from staff members at University College London. Healthy participants were eligible if they are age matched and did not suffer from health conditions, do not have bowel symptoms and are not on regular medication. Patients with the diagnosis of BCRP or CRC were excluded if they suffered from any other malignant condition. We used Kudo's pit pattern classification at endoscopy to exclude patients with hyperplastic and inflammatory polyps.^23^ We included those with Kudo's pit III‐V and histology confirming neoplasia (adenomatous polyps). Results from the above clinical tests were obtained from the hospital electronic health record system (EPIC: 2020 EPIC system Corporation, Verona, USA). This study received the appropriate ethical approval (UCL‐RFH Biobank REC reference: 16/WA/0289, Study reference: NC2016.007) and all participants provided written consent. Participants with cardiovascular and/or inflammatory conditions were excluded.

### 2.2 Microvesicle isolation by flow cytometry

Blood samples were collected in lavender EDTA vacutainer tubes (BD, Oxford, UK). Platelet poor plasma (PPP) was obtained by double centrifugation at 5000*g* for 5 min as previously described and stored in 100 μl aliquots at −80°C until required.^24^ MVs were then isolated from PPP after centrifugation at 17,000*g* for 1 h at 4°C. Annexin‐V conjugated to fluorescein isothiocyanate (FITC) that was diluted in annexin‐V buffer (BD Pharmingen) was used to identify total MVs using flow cytometry.^24^ 1.1 μm latex beads were used to set the upper threshold on forward scatter to distinguish maximum MVs size. MVs captured in this way were defined as annexin‐V+ co‐expressing‐specific cell surface markers, determined by using appropriate isotype control antibodies for each marker. MVs were enumerated in a standardised fashion by using the proportion of a fixed number of 3 μm latex beads counted and the volume of sample from which the MVs were analysed (Figure S3).

### 2.3 Flow cytometric analysis of cell surface receptors

To allow for multiple labelling of receptors simultaneously, MVs suspended in annexin‐V were further labelled with fluorescently conjugated antibodies (1:50 dilution) using different fluorochromes including phycoerythrin (PE), allophycyanin (APC) or APC‐Cy7. Human or mouse monoclonal antibodies used for staining MVs subpopulations were anti‐glycoprotein (A33; R&D Systems Abingdon, UK), anti‐carcinoembryonic antigen‐5 (CEA‐5; R&D Systems Abingdon, UK), anti‐leucine‐rich G protein‐coupled receptor 5 (LGR‐5; BD Pharmingen), anti‐Ephrin type‐B receptor 2 (EPHB2; BD Pharmingen), anti‐intercellular adhesion molecule (ICAM1, CD54; BD Pharmingen), anti‐platelet endothelial cell adhesion molecule (PECAM1, CD31; BD Pharmingen) and anti‐glycoprotein 9 (CD42a; BD Pharmingen). As the A33 protein, the stem cell marker LGR5, the adhesion molecule CEA‐5 and EPHB2 receptor are markers associated with CRC and their expression levels are elevated in cancer, MVs containing these receptors served to indicate tumour‐associated vesicles. MVs containing ICAM1, PECAM1 and CD42a represented vesicles that were associated with systemic inflammation. More specifically, CD31+ MVs represented a subpopulation that was derived from endothelial cells and platelets, CD42a + MVs represented those that were derived from platelets only, and the CD31+/CD42a‐ MVs represented endothelial‐derived vesicles. To distinguish non‐specific staining, isotype control antibodies anti‐mouse IgG1,k PE (BD Pharmingen), anti‐mouse IgG1 APC (R&D Systems Abingdon, UK) and anti‐mouse IgG1,k APC‐Cy7 (BD Pharmingen, New Jersey, USA) were used with protein: fluorochrome ratios equal to their associated fluorescence‐conjugated antibodies. The MV‐annexin‐V‐antibody suspensions in 96‐well plates were incubated in the dark at room temperature for 15 minutes, after which 200 μl of annexin V buffer was added to each well to neutralise the reaction. The plates were then read by a FACSArray BioAnalyzer™ flow cytometer (BD Biosciences, Oxford, UK). The gating was set by running unstained and isotype control‐stained cells through the cytometer and toggling the forward and side scatter and colour channels on logarithmic scales.

### 2.4 Statistical analysis

To determine the statistical power of the current sample size, post hoc power calculation was performed using G*Power version 3.1 (website: http://www.gpower.hhu.de/). For the given sample size statistical power was more than 95% for all *t*‐tests. Data were expressed as mean if parametric and median if non‐parametric. Missing data were not computed. Differences in means were assessed using the two‐way *t*‐test and Wilcoxon–Mann–Whitney test. Two‐way ANOVA test was used to examine the difference in mean of groups more than two. Logistic regression, factor analysis and correlation matrix were also performed to assess associations between variables. Area under the receiver operator curve (AUC), sensitivity, specificity, positive and negative predictive values were calculated when appropriate. All statistical analysis was carried out using Statistical Package for the Social Sciences” (IBM SPSS Statistics for Macintosh, version27, Armonk, NY: IBM Corp.) and GraphPad Prism (GraphPad Prism version 9 for MAC OSX, GraphPad Software, SanDiego, CA, www.graphpad.com). Statistical significance was regarded when P‐values were less than 5% (two sided).

## 3 RESULTS

### 3.1 Patient characteristics

Patients were similar in age and gender distribution between the groups. With regard to biochemical markers assessed in patients with BCRP and CRC, the blood levels of haemoglobin, lymphocytes, albumin, urea and creatinine were significantly lower in CRC patients by comparison with BCRP (Table 1). In contrast, other markers, including neutrophils and C‐reactive protein (CRP), were significantly higher in CRC patients by comparison with those diagnosed with BCRP. The predictive values of the routine bloods for CRC in comparison to BCRP are summarised in the supplementary material including AUC, cut‐off points, sensitivity, specificity, positive predictive value (PPV) and negative predictive value (NPV) (Tables S1) and logistic regression odds ratio (OR) (Table S2).

**TABLE 1:** Patients' characteristics

	Healthy control (*n* = 17)	BCRP (*n* = 16)	CRC (n = 19)	*p* value
Age in years: mean (SD)	56 (10)	62 (15)	61 (15)	0.43
Gender				0.34
Male	9 (53%)	12 (75%)	13 (68%)	
Female	8 (47%)	4 (25%)	6 (32%)	
Condition/Cancer stage	‐	Adenomatous polyps	Stage 1 (n = 1, 5%) Stage 2 (n = 3, 15%) Stage 3 (n = 15, 80%)	‐
Haemoglobin [g/L]: mean (SD)	‐	138 (10)	117 (22)	0.006
WCC [10^9^/L]: mean (SD)	‐	9 (3)	9 (3)	0.55
Neutrophils [10^9^/L]: mean (SD)	‐	5 (2)	7 (3)	0.02
Lymphocytes [10^9^/L]: mean (SD)	‐	2 (1)	1 (0.5)	0.003
Platelet [10^9^/L]: mean (SD)	‐	273 (55)	320 (168)	0.38
Albumin [10^9^/L]: mean (SD)	‐	45 (3)	37 (6)	0.0002
CRP [mg/L]: median (range)	‐	3 (0–67)	56 (0–295)	0.009
Creatinine [*μ*mol/L]: mean (SD)	‐	90 (20)	75 (20)	0.003
Urea [mmol/L]: mean (SD)	‐	6 (2)	4 (1)	0.0003

Abbreviations: BCRP, Benign colorectal polyps; CRC, colorectal carcinoma; CRP, C‐reactive protein; MD, moderately differentiated; NNP, non‐neoplastic Polyps; NP, neoplastic polyps; PD, poorly differentiated; SD, standard deviation; WCC, white cell count.

### 3.2 Plasma MVs in patients with BCRP and CRC

**FIGURE 1:**
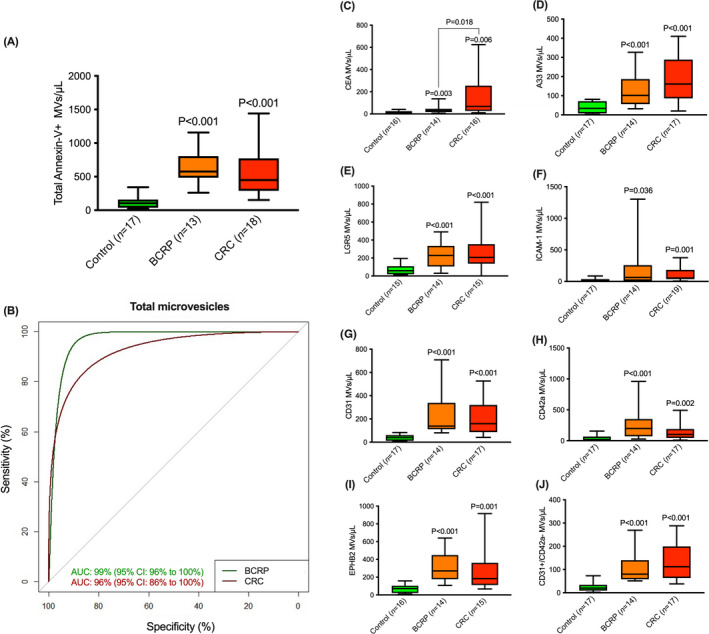
Total plasma MV (MVs) concentration in benign colorectal polyp (BCRP) and colorectal cancer (CRC) (A) and their diagnostic ability shown as area under the curve (AUC) (B). The plasma levels of MVs positive for potential markers of colorectal neoplasia in healthy controls, BCRP: and CRC including; (C) carcinoembryonic antigen (CEA); (D) A33; (E) leucine‐rich repeat containing G protein‐coupled receptor 5 (LGR5); (F) ephrin type‐B receptor 2 (EPHB2); (G) intercellular adhesion molecule 1 (ICAM‐1); (H) CD31; (I) CD42a; and (J) CD31+/CD42a‐

Total plasma MVs and sub‐populations positive for CEA, A33, LGR5, EPHB2, ICAM‐1, CD31, CD42a, CD31+/CD42a‐ (Figure 1) were significantly higher in patients diagnosed with BCRP and CRC in comparison to healthy controls (Table 2). AUC for total number of MVs (Figure 1B) and sub‐populations MVs demonstrated high and significant predictive value for BCRP and CRC (Table 3). Cut‐off value ≥211 of total plasma MVs/μl demonstrated a sensitivity of 100% (95% CI: 75%–100%) and a specificity of 88% (95% CI: 64%–99%) for the diagnosis of BCRP. This cut‐off point showed a likelihood ratio of 8.5, and positive and negative predictive values of 87% and 100%, respectively, for the diagnosis of BCRP. Similarly, a cut‐off ≥173 of total plasma MVs/μl demonstrated a sensitivity of 89% (95% CI: 65%–99%), a specificity of 88% (95% CI: 64%–99%) for the diagnosis of CRC. The likelihood ratio for this cut‐off point was 7.5. PPV and NPP were 89% and 88%, respectively for the diagnosis of CRC.

**TABLE 2:** The levels of selected colorectal cancer markers identified on MVs

	Plasma concentration (MVs/μl)			
	Healthy controls^†^	BCRP^†^	CRC^†^	*p* value
Total MVs: mean (SD)	124 (88)	654 (270)	558 (378)	<0.0001
CEA: median (range)	8 (1–40)	29 (7–136)	68 (10–625)	<0.0001
A33: mean (SD)	40 (29)	127 (87)	182 (121)	<0.0001
LGR5: mean (SD)	63 (54)	232 (147)	271 (207)	0.001
EPHB2: mean (SD)	71 (49)	324 (171)	276 (224)	0.0002
ICAM: median (range)	14 (2–87)	63 (5–1303)	54 (6–376)	0.008
CD31: mean (SD)	39 (28)	218 (175)	209 (150)	0.0003
CD42a: median (range)	18 (5–155)	198 (27–959)	102 (9–492)	0.0002
CD31+/CD42a‐: mean (SD)	25 (20)	110 (71)	135 (81)	<0.0001

Abbreviations: BCRP, benign colorectal polyps; CEA, carcinoembryonic antigen; CRC, colorectal; CRC, colorectal cancer; EPHB2, ephrin type‐B receptor 2; ICAM, intercellular adhesion molecule; LGR5, leucine‐rich repeat containing G protein‐coupled receptor 5; SD, standard deviation.

^†^ Sample size is variable please refer to Figure 1 for individual markers sample size.

**TABLE 3:** Diagnostic accuracy of component factors 1 and 2 for the diagnosis of BCRP and CRC

	AUC	95% CI	Cut‐off point (MVs/μl)	Sensitivity	Specificity	PPV	NPV	*p* value
BCRP								
Component factor 1	1	1–1	>761	100%	100%	93%	100%	<0.0001
Component factor 2	0.94	0.86–1	>222	100%	63%	70%	100%	<0.0001
CRC								
Component factor 1	0.95	0.88–1	>439	100%	56%	65%	100%	<0.0001
Component factor 2	0.93	0.84–1	>157	100%	50%	65%	100%	<0.0001

Abbreviations: AUC, area under the receiver operating curve; BCRP, benign colorectal polyps; CRC, colorectal cancer; CI, confidence interval; MVs, microvesicles; NPV, negative predictive value; PPV, positive predictive value.

A cut‐off value ≥144 of total plasma MVs/μl provided a 100% sensitivity for BCRP (95% CI: 75%–100%) and CRC (95% CI: 81%–100%). The specificity at this cut‐off value for BCRP and CRC was 59% (95% CI: 33%–82%: LR: 2.4) and 59% (95% CI: 33%–82%: LR: 2.4), respectively. For BCRP, PPV and NPV were 67% and 100%, respectively. For CRC diagnostic accuracy, PPV and NPV were 73% and 100%, respectively. Diagnostic values for total MVs and their sub‐populations for identifying patients with BCRP and CRC are summarised in supplementary appendix Tables S6 and S7.

CEA+ MVs were significantly higher in patients suffering from CRC compared to those with BCRP (Figure 1C and Table 2). A CEA+ positive MVs population was a distinguisher of CRC from BCRP (AUC = 75%; 95% CI = 57%–93%; *p* = 0.019). All other MVs sub‐populations including total plasma MVs showed no significant differences between BCRP and CRC.

### 3.3 Factor analysis

All the markers including total plasma MVs and MV sub‐populations were subjected to principal component analysis (PCA). Prior to performing PCA, the suitability of data for factor analysis was assessed by a correlation matrix and cluster grouping of the plasma MVs markers (Table S3). Dendrogram construction resulted three clusters: (1) CD31, total MVs, EPHB2, CD31+/CD42a‐ and CD42a; (2) LGR5, A33 and CEA; and (3) ICAM‐1 (Figure S3). Most correlation coefficients (Spearman's r) were above 0.3. The Kaiser‐Meyer‐Oklin value was 0.64, exceeding the recommended value of 0.6.^25^ Bartlett's Test of Sphericity^26^ was statistically significant (*p* < 0.001), supporting the factorability of the correlation matrix. PCA confirmed the presence of two components with eigenvalues exceeding 1, explaining 66% and 13% of the variance, respectively. Component factor 1 includes total plasma MVs, and MVs positive for CD31, CD42a, CD31+/CD42a‐, EPHB2, ICAM‐1 and LGR5. Similarly, component factor 2 included MVs positive for EPHB2, A33, CEA and LGR5 (Table S4). An inspection of the scree‐plot revealed a clear break after the second component (Figure S4). Using Cattell's scree test, it was decided to use the two component factors for further investigation (Table S5).^27^

There was a positive correlation between the two component factors (*r* = 0.47). Although for diagnostic propose component score coefficients can be used to compute the values of markers in the component factors for a particular patient, we chose to adopt a stringent method described by Grice.^28^ This method uses values generated from a pattern matrix (Table S4). It assigns a value of 1 to the coefficients for variables with loadings greater than 0.4 and zero to the coefficients for variables with loading equal to or less than 0.4. Values for component factors 1 and 2 and cut‐off points were computed using the Grice method.^28^ The following equation was used: *F1 = b11X1 + b12X2 + b13X3 +* etc. where value 1 was assigned to *b*‐coefficients for variables with loadings greater than 0.4, and 0 for variables with loadings equal to or less than 0.4.

**FIGURE 2:**
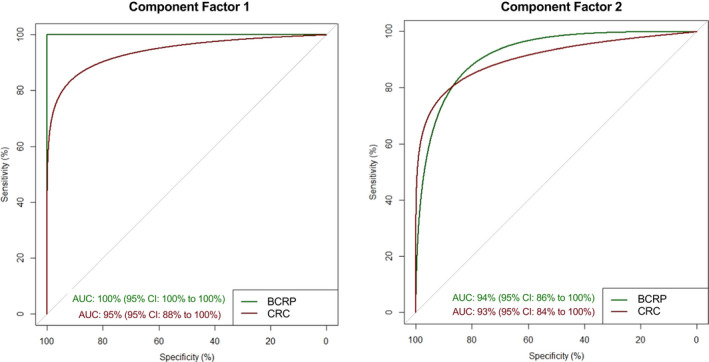
Area under the receiver operator curve (AUC) for the diagnosis of benign colorectal polyps (BCRP, line in green) and colorectal cancer (CRC, line in red) for component factors 1 (composed of: total plasma microvesicles (MVs) and sub‐populations positive for (CD31, CD42a, CD31+/CD42a‐, EPHB2, ICAM‐1 and LGR5) and component factor 2 (composed of: MV populations positive for EPHB2, A33 and LGR5). CI: confidence interval

Component factor 1 was able to significantly (*p* < 0.0001) diagnose BCRP and CRC with AUC of 100% (95% CI: 100%–100%) and 95% (95% CI: 88%–100%), respectively. Similarly, component factor 2 was able to significantly (*p* < 0.0001) diagnose BCRP and CRC with AUC of 94% (95% CI: 86%–100%) and 93% (95% CI: 84%–100%), respectively (Table 3 and Figure 2).

## 4 DISCUSSION

We found a peripheral blood biomarker that can predict the diagnosis of colorectal neoplasia with high accuracy. With little overlap, total plasma annexin‐V+ MVs were significantly high in patients with BCRP, and CRC compared to healthy controls. Furthermore, biomarkers that were described for their prognostic, but often poor diagnostic values in colorectal neoplasia, such as CEA,^14^ EPHB2,^15^ ICAM‐1,^16^ LGR5,^17^ A33,^18^ CD31^19^ and CD42a (a platelet membrane glycoprotein),^29^ can be used to isolate MV populations that we discovered to have exceptionally high predictive value. Most importantly, MVs were highly predictive of BCRP, yielding an impressively positive predictive value of 93%. The clinical implications of our findings are extensive in that the MVs as a screening tool can provide a good clinical utility. A small volume of 100 μl of platelet poor plasma is sufficient to perform a MV test (MVT), which can potentially be isolated from a finger‐prick capillary blood sample. With high predictive value as described and improved compliance, MVT can cut the chain of costly and invasive investigations at a time when healthcare resources are overstretched. This novel finding provides a unique opportunity for developing a platform for early cancer diagnosis that is universally acceptable across different socio‐demographic groups, with potential for saving cost, and improving survival and quality of life.

To date, all the literature on EVs as biomarkers in CRC is focused on exosomes.^11^ After a recent extensive literature search, we could not find a single study that identified the true MVs or apoptotic bodies as biomarkers for CRC. MVs are biogenically and structurally different from exosomes and apoptotic bodies. Nevertheless, there are a lot of lessons learnt from studying exosomes as biomarkers. Here follows a brief synopsis of exosomes as biomarkers in relation to CRC. Some studies characterised the miRNA extracted from exosomes,^30^ and a few examined their protein profile.^31^ Previously, extracellular matrix metalloprotease (CD147) positive exosomes were found to be significantly higher in patients suffering from CRC in comparison to healthy controls.^32^ The area under the curve (AUC) was high (93%) for CD147+ exosomes, however, total plasma exosomes showed poor predictive value (AUC = 63%). Other studies characterised exosomes from CRC‐derived cell lines in culture, although purification of exosomes from the supernatant is challenging due to their small size (30–150 nm) and the need for ultracentrifugation (100,000 g). One study did find A33 and epithelial cell adhesion molecule (EpCAM) to be highly expressed in exosomes shed by a CRC cell line (LIM1863).^33^, ^34^ Protein expression profile of exosomes from lymph nodes metastatic CRC cell lines (SW480 and SW620) demonstrated a distinct protein signature that plays a role in cancer metastasis.^35^ Another study purified exosomes from ascitic fluid of patients with CRC and reported a distinguished protein profile.^36^ However, to date we were unable to find any studies that examined the clinical application of these markers. Although, some authors referred to their studied particles as MVs, their purification methods and size (30–150 nm) suggest that they are in fact exosomes. These studies did not assess annexin‐V+ MVs, but rather used the classic markers of exosomes (CD9, CD63 and CD81), a method that would likely omit a large population of MVs.^37^

Specific centrifugation method and annexin‐V staining are required to detect MVs. Annexin‐V is a member of the phospholipid‐binding annexin family.^38^ It binds with high affinity to phosphatidylserine (PS),^39^ a phospholipid that is normally retained in the inner leaflet of the plasma membrane, and is well known as a marker of apoptotosis.^40^ Upon receipt of apoptotic signals, PS is transported from the inner to the outer leaflet of the plasma membrane.^41^ The exteriorisation of PS to the outer leaflet of the plasma membrane is a well‐recognised phenomenon relating to tumour microenvironment, due to hypoxia and the presence of oxygen radicals.^42^ Indeed, abundant PS expression was described in malignant cells and tumour‐associated vascular endothelial cells.^43^, ^44^ Although apoptotic bodies are also isolated using annexin‐V,^45^ the size gaiting to purify them is different to that of MVs. We believe that because of their small size and biogenesis, annexin‐V+ MVs may be ideal candidates to identify MVs population with surface biomarkers related to colorectal neoplasia.

The biomarkers identified here have been described before, but in different context. Serum CEA level is one of the most widely used biomarkers of CRC recurrence.^46^ Its role in early cancer detection is limited due to low sensitivity and specificity, it is found to be elevated in approximately 47% of CRC patients.^47^ Similarly, EPHB2, a class of transmembrane ligand families of ephrins, is expressed in approximately 60% of CRC tissue samples and cell lines and has been associated with longer mean duration of survival.^15^ ICAM‐1, is a co‐stimulator that binds to lymphocyte function‐associated antigen 1 on the surface of T cells.^48^ ICAM‐1 is known to be a prerequisite for leucocyte trafficking thorough endothelial and epithelial barrier and therefore mediates host defence.^48^ Interestingly, ICAM‐1 expression was found to be significantly associated with CRC that displays microsatellite instability, with a favourable prognosis.^16^ On the other hand, a meta‐analysis by Jiang et al. showed that high expression of LGR5 was associated with poor survival in colorectal cancer.^17^ Although the function of the glycoprotein A33 is unclear, it has been recognised as a specific marker of colonic epithelium and cancer.^49^ A33 is currently being developed to target colon cancer cells for immunotherapy.^50^ Other endothelial marker involved in cancer microenvironment and angiogenesis, such as CD31 and CD 42a were also assessed. High expression of CD31 has been associated with worse patients' survival in CRC.^19^ CD42a is a platelet surface membrane glycoprotein that functions as a receptor for von Willebrand factor and is used to mark platelet‐related extracellular vesicles with potential role in promoting cancer cell survival.^20^ Although the role of these biomarkers in CRC progression is beyond the scope of this study, the ability to characterise CRC microenvironment in individual cases using MVs can have wide implications in personalised oncotherapy. MVs subpopulations could provide a better preoperative workup of individual patients, enabling accurate prognostic predictors and informing a more effective therapeutic strategies.

Unlike previous research into MVs in CRC, our study offers a novel approach that provides a significant leap forward in the field. We found an outlet of a previously described method for isolating MVs from endothelial cells,^24^ using annexin‐V,^24^, ^37^ to repurpose MVs as diagnostic markers. Our study design and findings are strengthened by their clinical relevance and translational potential in the clinic. Inherently, the results may be weakened by confounding factors, lack of randomisation, lack of comparison to a control group of patients undergoing colonoscopy with similar symptoms. However, as a proof‐of‐concept, we included a healthy controls group of participants without known medical conditions but with similar demographics. The small sample size (<100 participants/group) is a limitation to this study implying a high probability of a 5% over‐ or under‐estimation of the predictive values in the results.^51^ However, even if the results are overestimated by 5%, the proposed MVT is still high. Moreover, a part from CEA, all other markers identified were not significant discriminators between patients with BCRP and CRC. Nevertheless, we identified some routine blood tests that can significantly distinguish BCRP from CRC, results are summarised in the supplementary material (Table S1 and S2). These could be used to prioritise further investigations for those whose MVT is positive, and not in isolation as they may result in high false positive rate. Unfortunately, we did not collect routine blood samples from the healthy control and did not measure CEA serum level in participating patients. This is a limitation that need to be addressed in a validation study. In a clinical context, predicting BCRP is as important as predicting CRC, because BCRP often progresses to become cancer,^52^ therefore, their identification and surgical excision is a key to prevent CRC.^53^ We have yet to determine how sensitive and specific this test will be when comparing BCRP and CRC to other bowel diseases.

Going forward, manufacturing a home blood collection kit harnessing a finger‐prick and carrying out a clinical trial will be important for progressing this research into a true clinical application. To inform any future clinical application, it will be necessary to understand both the compliance and cost effectiveness. It is possible that the finger‐prick capillary sampling is not as optimal as peripheral blood for MVs purification. Even if this strategy fails due to finger‐prick capillary not providing a sufficient sample, peripheral blood MVT could be part of the UK NHS health check programme, which is offered to participants age between 40 and 74 years old.^54^ Although this programme could be a useful vehicle for MVT, currently the compliance for participation in people with an average age of 55 years is low at 65%.^55^ Understanding the test and screening programme costs is one element of cost effectiveness. A full economic evaluation of the additional cost and health outcomes of the MVs as a bowel cancer screening test will be important for informing future research and clinical utility.

**FIGURE 3:**
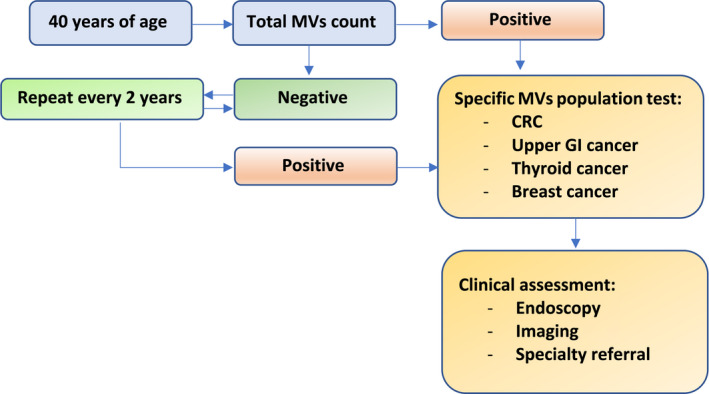
Microvesicles (MVs) test as a screening tool visionary pathway. CRC: colorectal cancer. GI: gastrointestinal

A MVT could achieve high compliance and affordability. Thus, we predict it would allow us to lower the age of the screening programme to 40 years. A hypothetical screening pathway would involve a capillary blood sample to measure the total plasma MVs concentration. If this is positive, patients should then be invited to undergo a specific MVs population test. Further research is required to ascertain the specificity of MVs to disease. Subsequently, the same methods can be applied to identify specific markers to other tumours (Figure 3). A specific MVs population test could be a second step where patients are streamlined for further invasive investigations depending on the test results and the clinical assessment. Whether to use total plasma MVs count or the described component factors as a screening test depends on the cost and feasibility. The cut‐off points will be used to identify positive from negative cases. Although different flow cytometer analysers may give variable cut‐off points that would require adjustments. We would like to emphasise that this study is limited by its sample size and require validation on a larger cohort of patients and comparable controls to verify and realise the potential of MVs as biomarkers for early detection of CRC.

## 5 CONCLUSION

MVs as biomarkers provide a platform for a simple and potentially effective screening tool, which yields a breakthrough in the early and accurate detection of CRC. MVs as markers of disease in general may have far reaching implications that extend to early detection of other diseases and inform their true incidence, thus improving our understanding of pathogenic mechanisms and advancing novel diagnostic modalities.

## CONFLICT OF INTEREST

This work has been filed for patent in the UK (Intellectual Property Office Reference: N420839GB); ME, RG, RC, ML, and LC are inventors on this patent.

## AUTHOR CONTRIBUTIONS

Conceptualization: MMRE, RG, RC, ML, LC. Methodology: MMRE, RG, KF. Investigation: MMRE, RG. Visualisation: MMRE, RG. Funding acquisition: MMRE, RC. Project administration: MMRE. Supervision: MMRE, RC, ML, LC. Writing – original draft: MMRE. Writing – review & editing: MMRE, RG, KF, RC, PL, ML, LC.

## ETHICAL STATEMENT

The authors are accountable for all aspects of this work in ensuring that questions related to the accuracy or integrity of any part of this work are appropriately investigated and resolved. This study was conducted in accordance with the Declaration of Helsinki (as revised in 2013). This study received the appropriate ethical approval (UCL‐RFH Biobank REC reference: s16/WA/0289, Study reference: NC2016.007) and all participants provided written consent.

## Supporting information


Figure S1

Figure S2

Figure S3

Figure S4

Data S1

## Data Availability

The data that support the findings of this study are available upon request from the corresponding author. The data are not publicly available due to privacy or ethical restrictions.
