# Supplementary figures and images for: The role of microvesicles as biomarkers in the screening of colorectal neoplasm
Source: Cancer Med. 2022 Mar 27;11(15):2957–68. doi: 10.1002/cam4.4664 (PMC9359869; doi:10.1002/cam4.4664)

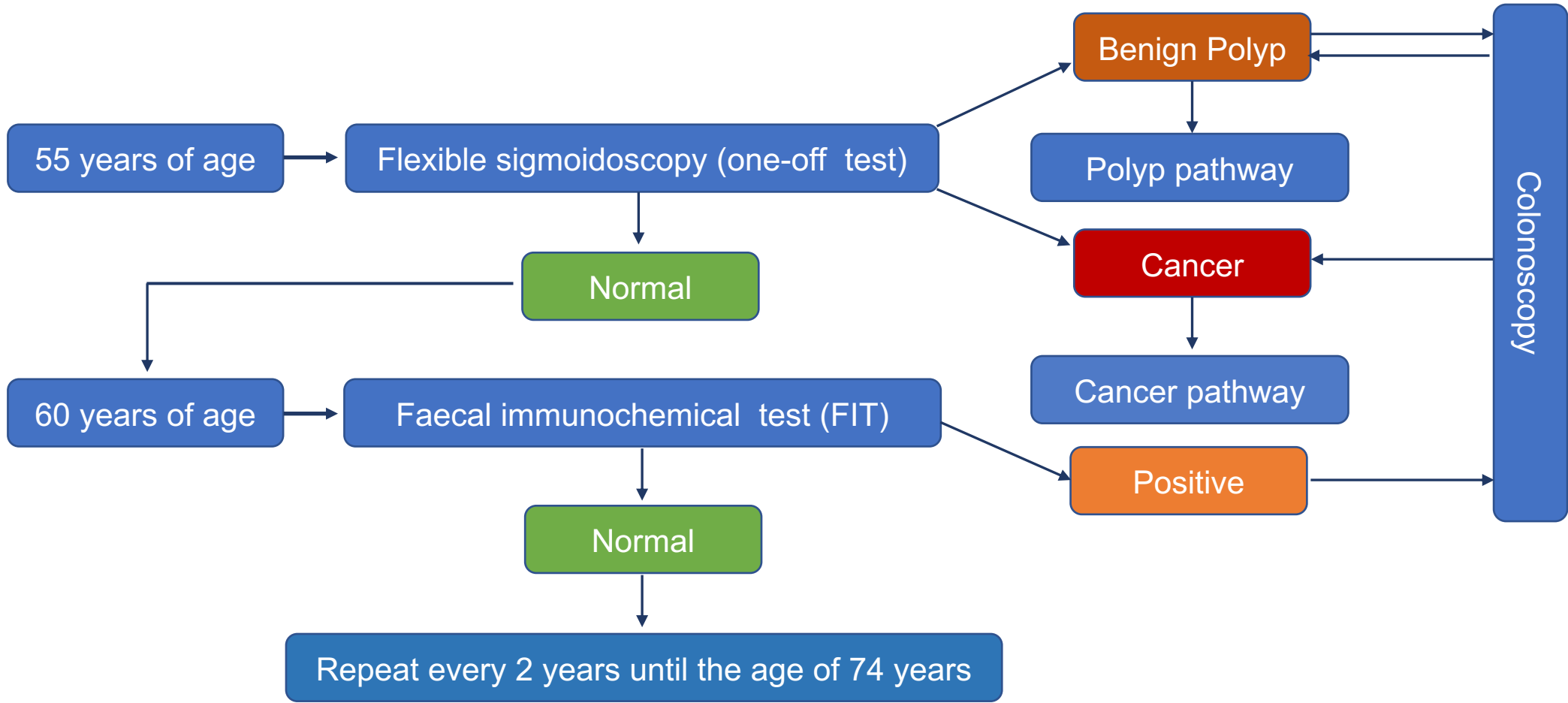

Supplement: Supplementary file 1 — Figure S1 [file CAM4-11-2957-s005.pdf]

(A)

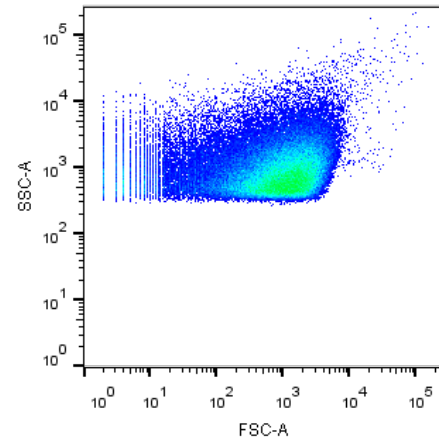

(B)

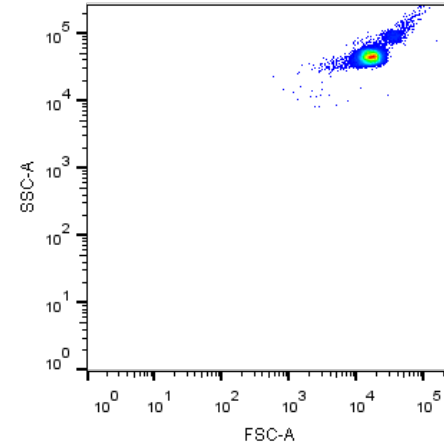

(C)

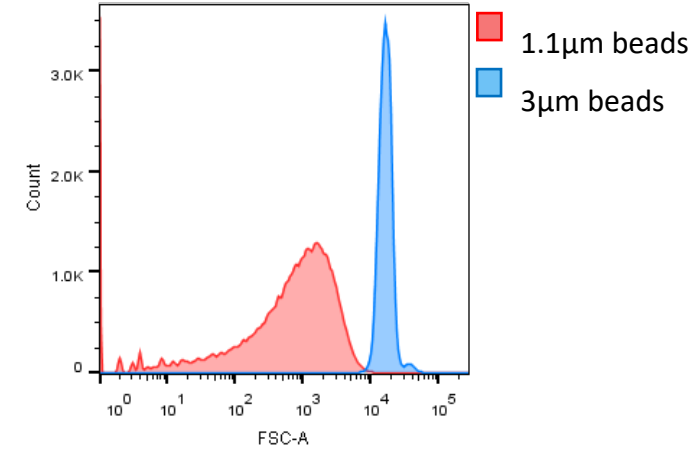

(D)

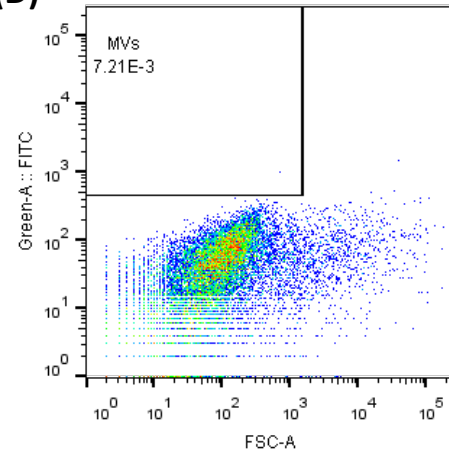

(E)

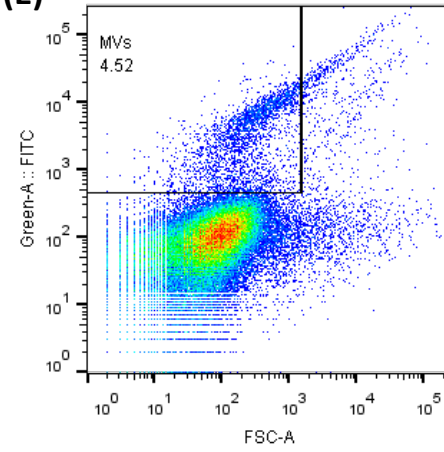

(F)

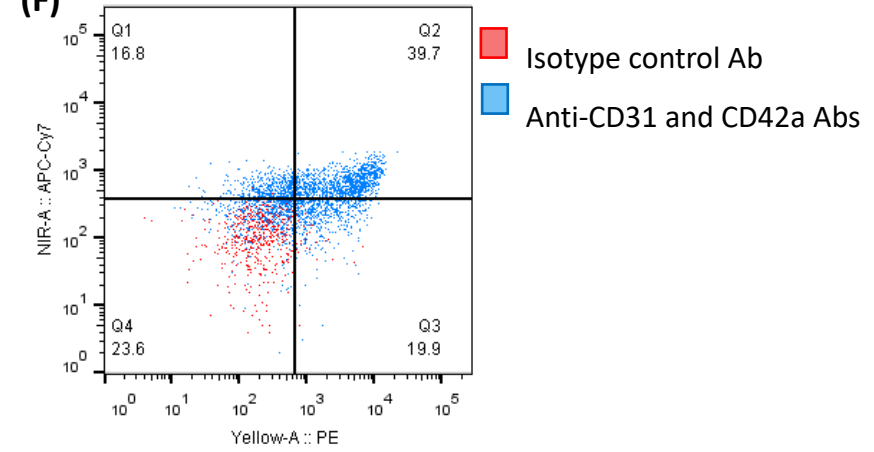

Supplement: Supplementary file 2 — Figure S2 [file CAM4-11-2957-s002.pdf]

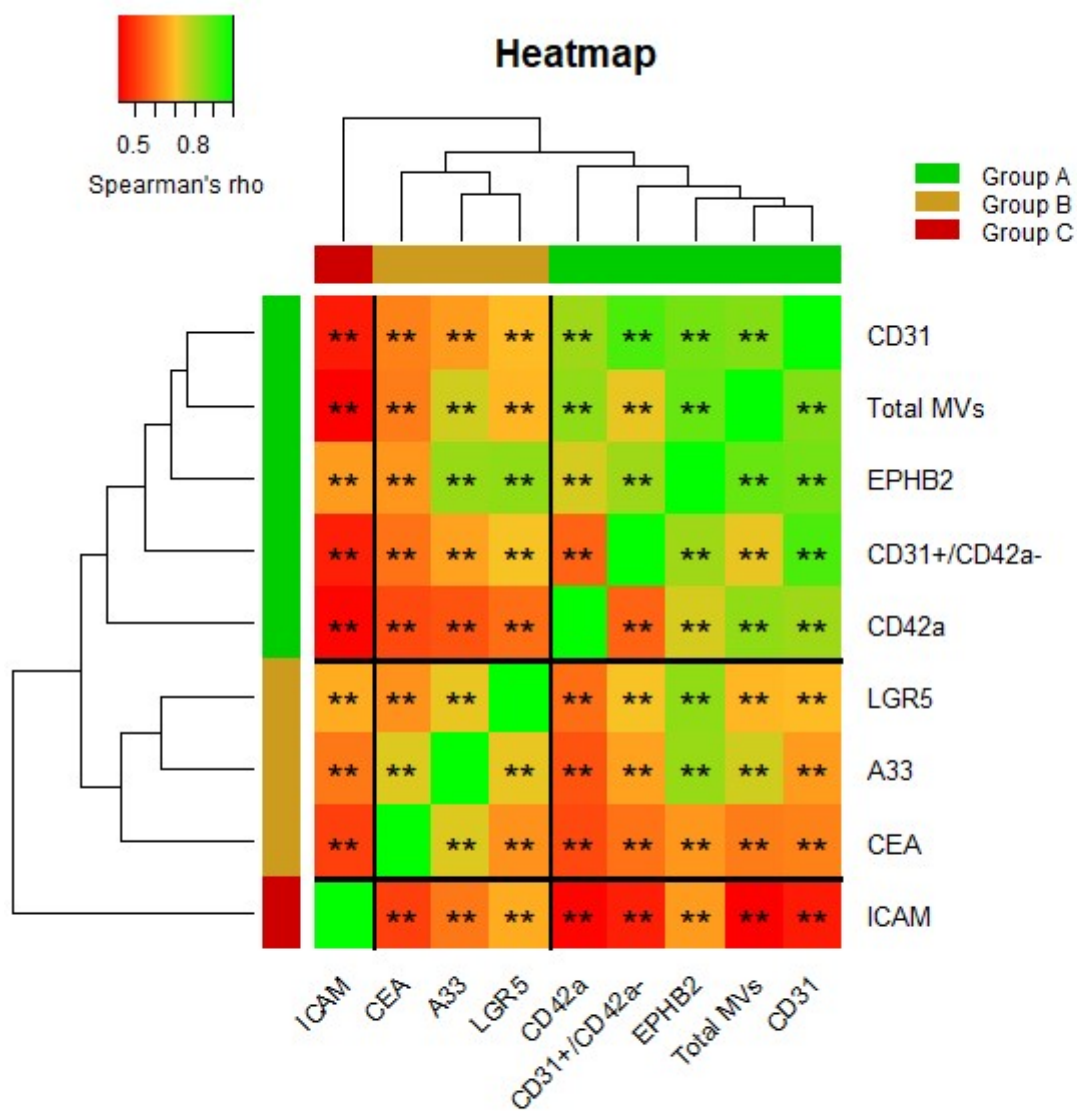

Supplement: Supplementary file 3 — Figure S3 [file CAM4-11-2957-s001.pdf]

**Scree Plot**

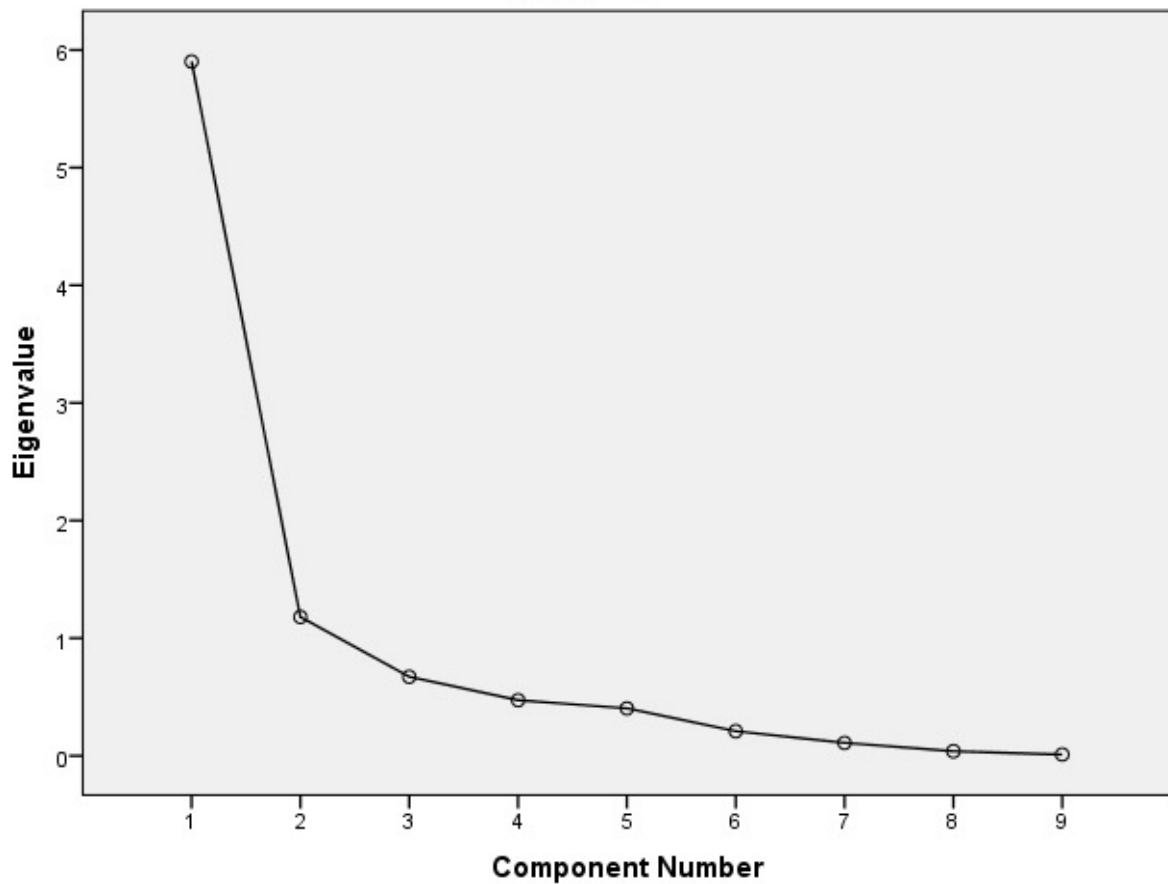

Supplement: Supplementary file 4 — Figure S4 [file CAM4-11-2957-s003.pdf]
