# Supplementary material for: The role of microvesicles as biomarkers in the screening of colorectal neoplasm
Source: Cancer Med. 2022 Mar 27;11(15):2957–68. doi: 10.1002/cam4.4664 (PMC9359869; doi:10.1002/cam4.4664)
Supplement: Supplementary file 5 — Data S1 [file CAM4-11-2957-s004.docx]

# Supplementary Appendix

[Supplementary Appendix 1](#_Toc72276261)

[Figures Supplementary Material 2](#_Toc72276262)

[Fig. S1. 2](#_Toc72276263)

[Fig. S2. 3](#_Toc72276264)

[Fig. S3 4](#_Toc72276265)

[Fig. S4. 5](#_Toc72276266)

[Tables Supplementary Material 6](#_Toc72276267)

[Table S1. 6](#_Toc72276268)

[Table S2. 7](#_Toc72276269)

[Table S3. 8](#_Toc72276270)

[Table S4. 9](#_Toc72276271)

[Table S5. 10](#_Toc72276272)

[Table S6. 11](#_Toc72276273)

[Table S7. 12](#_Toc72276274)

# Figures Supplementary Material

## Fig. S1.

| 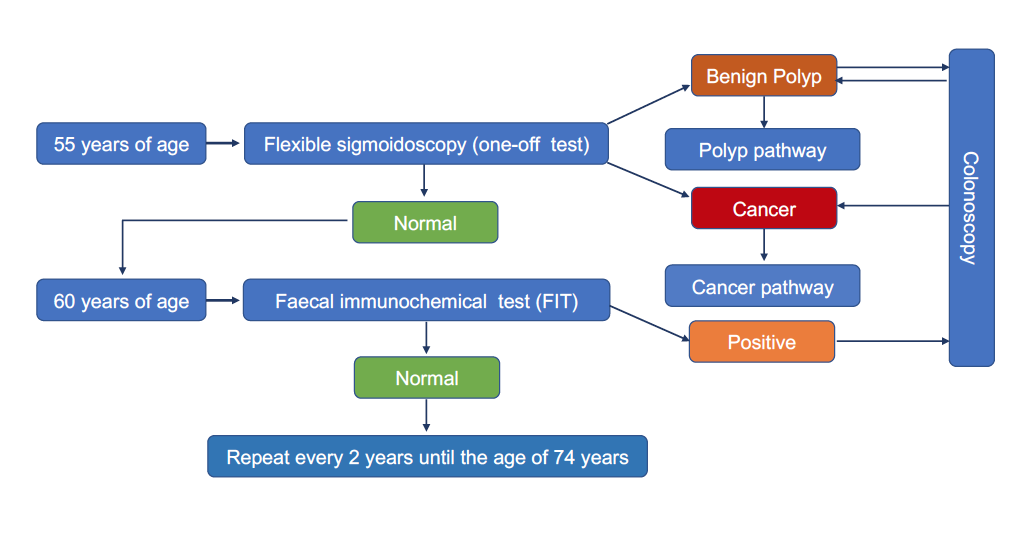  **Fig. 1S. Bowel cancer screening program in the UK summary of the current pathway.** |
| --- |

## Fig. S2.

| 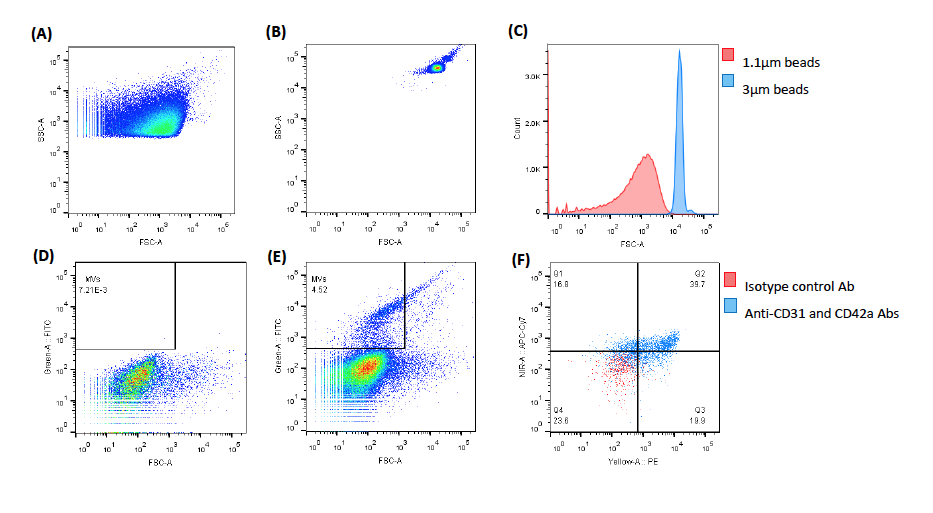  Fig. S3. Flow cytometric analysis of microvesicles and latex microbeads. (A-C) Dot plots and histograms of 1.1μm (red) and 3μm latex beads (blue) used for MV size gating and enumeration, respectively. (D) Unstained microvesicles and 1.1μm beads were used to determine the gating of Annexin V-FITC+ MVs from all samples. (E) Microvesicles derived from a benign colorectal polyps patient gated by Annexin V+ and size. (F) Isotype control antibodies were used to determine the gating for identifying microvesicles population (red). Distribution of microvesicles from a benign colorectal polyps patient is shown depicting the identification of endothelial (CD31+/CD42a-) and platelet (CD42a+) derived microvesicles population (blue). |
| --- |

## Fig. S3

| 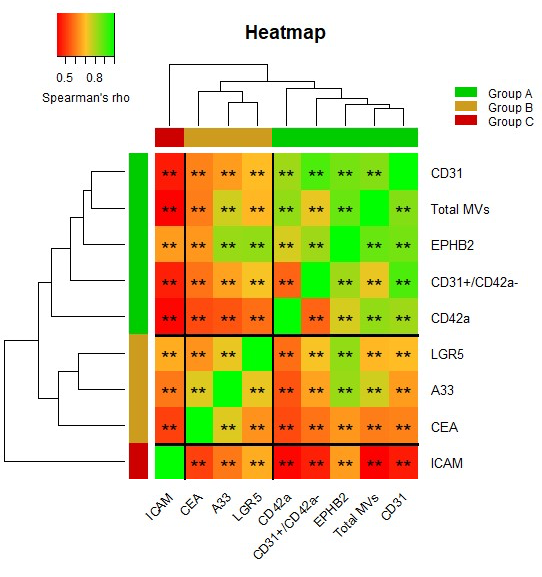  Fig. S3. Correlation matrix, heatmap showing significance of Spearman’s rho. Dendogram showing markers clustering into three groups: A) CD31, total microvesicles, EPHB2, CD31+/CD42a- and CD42a; B) LGR5, A33, and CEA; C) ICAM. **: *p*<0.01. |
| --- |

## Fig. S4

| 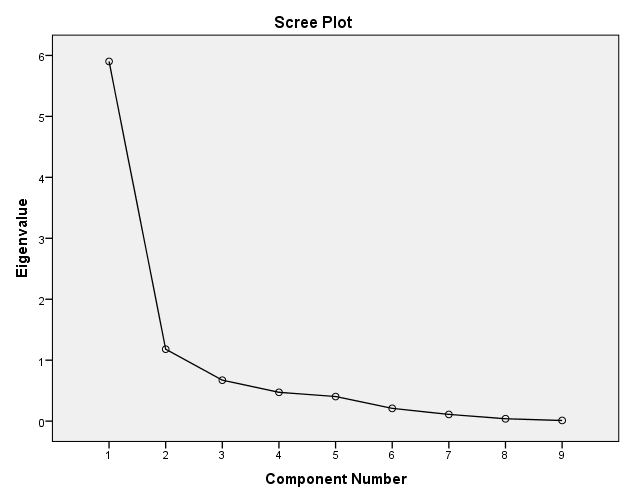  **Fig. S4. Scree plot showing 2 component factors appropriateness for further investigation.** |
| --- |

# Tables Supplementary Material

## Table S1.

| Table S1. Diagnostic accuracy of routine blood and MVs CEA+ for distinguishing between patients suffering from BCRP and CRC. The control group are results from patients with BCRP. | | | | | | | | |
| --- | --- | --- | --- | --- | --- | --- | --- | --- |
|  | **AUC** | **95% CI** | **Cut-off point** | **Sensitivity** | **Specificity** | **PPV** | **NPV** | ***p* value** |
| **Haemoglobin (g/L)** | 0.87 | 0.73 to 1 | <124.5 | 68% | 100% | 100% | 63% | 0.001 |
| **Neutrophils (10^9^/L)** | 0.76 | 0.59 to 0.93 | >7.7 | 47% | 100% | 100% | 50% | 0.02 |
| **Lymphocytes (10^9^/L)** | 0.81 | 0.64 to 0.97 | <1.16 | 47% | 100% | 100% | 50% | 0.008 |
| **Albumen (g/L)** | 0.9 | 0.78 to 1.01 | <39.5 | 63% | 100% | 100% | 61% | 0.0004 |
| **CRP (mg/L)** | 0.85 | 0.68 to 1.03 | >72.75 | 47% | 100% | 100% | 38% | 0.01 |
| **Urea (mmol/L)** | 0.85 | 0.71 to 1 | <3.85 | 58% | 100% | 100% | 56% | 0.002 |
| **Creatinine (*μ*mol/L)** | 0.75 | 0.69 to 0.98 | <63.5 | 53% | 100% | 100% | 55% | 0.003 |
| **CEA (MVs/µL)** | 0.75 | 0.57 to 0.93 | >221 | 25% | 100% | 100% | 54% | 0.02 |
| AUC: area under the receiver operating curve. BCRP: benign colorectal polyps. CEA: carcinoembryonic antigen. CRC: colorectal cancer. CRP: C-reactive protein. CI: confidence interval. MVs: microvesicles. NPV: negative predictive value. PPV: positive predictive value. | | | | | | | | |

## Table S2.

| Table S2. Logistic regression analysis to assess the probability of CRC from routine blood tests and MVs positive for CEA showing OR and significance. | | | | | | |
| --- | --- | --- | --- | --- | --- | --- |
|  | **OR** | **95% CI** | | ***B*** | ***Constant*** | ***p* value** |
|  |  | **Lower** | **Upper** |  |  |  |
| **Haemoglobin (g/L)** | 0.93 | 0.88 | 0.99 | -0.71 | 9.59 | 0.02 |
| **Neutrophils (10^9^/L)** | 1.17 | 0.89 | 1.54 | 0.16 | -0.46 | 0.27 |
| **Lymphocytes (10^9^/L)** | 0.21 | 0.60 | 0.75 | -1.56 | 3.50 | 0.01 |
| **Albumin (g/L)** | 0.62 | 0.42 | 0.90 | -0.49 | 20.87 | 0.01 |
| **CRP (mg/L)** | 1.05 | 0.99 | 1.09 | 0.04 | -0.35 | 0.06 |
| **Urea (mmol/L)** | 0.33 | 0.14 | 0.79 | -1.10 | 5.86 | 0.01 |
| **Creatinine (*μ*mol/L)** | 0.94 | 0.90 | 0.99 | -0.06 | 5.20 | 0.01 |
| **CEA (MVs/µL)** | 1 | 1 | 1 | 0 | -0.86 | 0.14 |
| CEA: carcinoembryonic antigen. CRC: colorectal cancer. CRP: C-reactive protein. CI: confidence interval. MVs: microvesicles. | | | | | | |

## Table S3.

| Table S3. Correlation matrix of total microvesicles and subpopulations positive for colorectal neoplasia related markers. | | | | | | | | |
| --- | --- | --- | --- | --- | --- | --- | --- | --- |
|  | **CEA** | **A33** | **LGR5** | **EPHB2** | **ICAM** | **CD31** | **CD42a** | **CD31+/CD42a-** |
| **Total microvesicles** | .31 | .65 | .55 | .69 | .51 | .78 | .78 | .66 |
|  | **CEA** | .66 | .53 | .40 | .40 | .26 | .23 | .28 |
|  |  | **A33** | .78 | .83 | .77 | .66 | .59 | .62 |
|  |  |  | **LGR5** | .89 | .52 | .54 | .41 | .62 |
|  |  |  |  | **EPHB2** | .58 | .72 | .65 | .71 |
|  |  |  |  |  | **ICAM** | .63 | .64 | .51 |
|  |  |  |  |  |  | **CD31** | .88 | .87 |
|  |  |  |  |  |  |  | **CD42a** | .56 |

## Table S4.

| Table S4. Pattern and structure matrix for principal component analysis with Oblimin rotation with Kaiser normalisation of two factor solution | | | | | |
| --- | --- | --- | --- | --- | --- |
|  | **Pattern coefficients** | | **Structure coefficients** | | **Communalities** |
|  | **Component 1** | **Component 2** | **Component 1** | **Component 2** |  |
| **CD31** | 1.002 |  | .964 | .389 | 0.935 |
| **CD42a** | .964 |  | .896 | .307 | 0.820 |
| **Total microvesicles** | .862 |  | .870 | .420 | 0.757 |
| **CD31+/CD42a-** | .799 |  | .838 | .456 | 0.707 |
| **EPHB2** | .618 | .432 | .820 | .721 | 0.818 |
| **ICAM** | .541 | .348 | .703 | .601 | 0.589 |
| **A33** |  | .940 | .741 | .878 | 0.911 |
| **CEA** | .305 | .700 |  | .860 | 0.762 |
| **LGR5** | .423 | .681 | .632 | .842 | 0.782 |

## Table S5.

| Table S5. Component score coefficient matrix | | |
| --- | --- | --- |
|  | **Component 1** | **Component 2** |
| **Total microvesicles** | .209 | -.055 |
| **CEA** | -.112 | .465 |
| **A33** | .053 | .296 |
| **LGR5** | .023 | .314 |
| **EPHB2** | .119 | .162 |
| **ICAM** | .107 | .128 |
| **CD31** | .251 | -.112 |
| **CD42a** | .246 | -.140 |
| **CD31+/CD42a-** | .189 | -.019 |

## Table S6.

| **Table S6. Predicting benign polyps** | | | | | | |
| --- | --- | --- | --- | --- | --- | --- |
| **Biomarker** | **AUC** | **95% CI** | **Cut-off point** | **Sensitivity** | **Specificity** | ***p*-value** |
| **Total MVs** | 0.99 | 0.96-1.00 | >244 MVs/µL | 100% | 93% | *<0.0001* |
| **CEA** | 0.84 | 0.71-0.98 | >6.37 MVs/µL | 100% | 40% | *0.001* |
| **A33** | 0.68 | 0.48-0.88 | - | - | - | *0.08* |
| **LGR5** | 0.93 | 0.85-1.00 | >28.4 MVs/µL | 100% | 60% | *<0.0001* |
| **EPHB2** | 0.89 | 0.78-1.00 | >1.07 MVs/µL | 100% | 47% | *<0.0001* |
| **ICAM-1** | 0.79 | 0.63-0.96 | >3.6 MVs/µL | 100% | 13% | *0.006* |
| **CD31** | 0.99 | 0.97-1.00 | >72 MVs/µL | 100% | 87% | *<0.0001* |
| **CD42a** | 0.90 | 0.79-1.00 | >25 MVs/µL | 100% | 47% | *<0.0001* |
| **CD31+/CD42a-** | 0.98 | 0.95-1.00 | >44 MVs/µL | 100% | 87% | *<0.0001* |
| **CK20** | 0.66 | 0.47-0.86 | - | - | - | *0.12* |
| **CK7** | 0.90 | 0.78-1.00 | >11 MVs/µL | 100% | 33% | *<0.0001* |
| **CD20+/CD7-** | 0.39 | 0.19-0.60 | - | - | - | *0.30* |
| **HLA-DR+** | 0.84 | 0.68-0.99 | >16 MVs/µL | 100% | 7% | *0.001* |
| **CD147** | 0.84 | 0.70-0.99 | >7.4 MVs/µL | 100% | 13% | *0.001* |
| AUC: area under the receiver operating curve. BCRP: benign colorectal polyps. CRC: colorectal cancer. CI: confidence interval. MVs: microvesicles. | | | | | | |

## Table S7.

| **Table S7. Predicting colorectal cancer** | | | | | | |
| --- | --- | --- | --- | --- | --- | --- |
| **Biomarker** | **AUC** | **95% CI** | **Cut-off point** | **Sensitivity** | **Specificity** | **P value** |
| **Total MVs** | 0.92 | 0.83-1.00 | >145 MVs/µL | 100% | 40% | *<0.0001* |
| **CEA** | 0.96 | 0.90-1.00 | >10 MVs/µL | 100% | 60% | *<0.0001* |
| **A33** | 0.87 | 0.75-0.99 | >13 MVs/µL | 100% | 13% | *<0.0001* |
| **LGR5** | 0.87 | 0.76-0.98 | >19 MVs/µL | 96% | 53% | *<0.0001* |
| **EPHB2** | 0.84 | 0.72-0.97 | >52 MVs/µL | 100% | 13% | *<0.0001* |
| **ICAM-1** | 0.90 | 0.80-1.00 | >12 MVs/µL | 96% | 47% | *<0.0001* |
| **CD31** | 0.97 | 0.93-1.00 | >38 MVs/µL | 100% | 60% | *<0.0001* |
| **CD42a** | 0.86 | 0.75-0.98 | >9 MVs/µL | 100% | 20% | *<0.0001* |
| **CD31+/CD42a-** | 0.95 | 0.87-1.00 | >38 MVs/µL | 96% | 87% | *<0.0001* |
| **CK20** | 0.76 | 0.61-0.92 | >8 MVs/µL | 100% | 47% | *0.006* |
| **CK7** | 0.93 | 0.85-1.00 | >16 MVs/µL | 100% | 47% | *<0.0001* |
| **CD20+/CD7-** | 0.75 | 0.60-0.91 | >4 MVs/µL | 100% | 33% | *0.009* |
| **HLA-DR+** | 0.89 | 0.80-0.99 | >26 MVs/µL | 100% | 27% | *<0.0001* |
| **CD147** | 0.85 | 0.73-0.97 | >17 MVs/µL | 96% | 27% | *<0.0001* |
| AUC: area under the receiver operating curve. BCRP: benign colorectal polyps. CRC: colorectal cancer. CI: confidence interval. MVs: microvesicles. | | | | | | |
